# Self‐compassion influences the caring behaviour and compassion competence among saudi nursing students: A multi‐university study

**DOI:** 10.1002/nop2.848

**Published:** 2021-03-10

**Authors:** Nahed Alquwez, Jonas Preposi Cruz, Abdulellah Al Thobaity, Joseph Almazan, Hawa Alabdulaziz, Farhan Alshammari, Monirah Albloushi, Regie Tumala, Abdulrahman Albougami

**Affiliations:** ^1^ Nursing Department College of Applied Medical Sciences Shaqra University Al Dawadmi Saudi Arabia; ^2^ Nursing Department Applied Medical Sciences Taif University Taif Saudi Arabia; ^3^ School of Medicine Nazarbayev University Nur‐Sultan Kazakhstan; ^4^ Faculty of Nursing King Abdulaziz University Jeddah Saudi Arabia; ^5^ College of Nursing University of Hail Hail Saudi Arabia; ^6^ College of Nursing King Saud University Riyadh Saudi Arabia; ^7^ Nursing Department College of Applied Medical Sciences Majmaah University Al Majmaah Saudi Arabia

**Keywords:** caring behaviour, compassion competence, nursing students, Saudi Arabia, self‐compassion

## Abstract

**Aim:**

This research investigated the perceptions of Saudi nursing students regarding self‐compassion and examined its influence on their caring behaviour and compassion competence.

**Design:**

This is a quantitative and cross‐sectional study.

**Methods:**

This multiuniversity study surveyed a convenience sample of 961 nursing using the Self‐compassion Scale, Compassion Competence Scale and Caring Behavior Inventory‐16 version. Standard multiple analysis was performed separately for caring behaviour and compassion competence as dependent variables. For both models, the self‐compassion dimensions and the demographic variables were considered predictor variables.

**Results:**

The students reported their self‐compassion moderately, with mindfulness receiving the highest mean and self‐judgment receiving the lowest mean. Significant differences were observed on self‐compassion of students between universities. Caring behaviour and compassion competence were also rated moderately by the students. “Self‐kindness” and “common humanity” were identified as significant predictors of caring behaviour, whereas “self‐kindness,” “self‐judgment,” “common humanity” and “mindfulness” were significant predictors of compassion competence.

## 1 INTRODUCTION

Caring and compassion are important and intertwining concepts in the nursing profession. Caring is a fundamental concept in nursing practice. Compassion is a virtue and a critical component of nursing care because it serves as the human and moral aspect of care (Su et al., 2020). Compassionate care is the “union of empathy related to and interacting with a nurse's desire to alleviate patients’ suffering, addressing individualized care needs, using therapeutic communication and promoting mutual benefits with their patients” (Su et al., 2020, p. 491). Hence, future nurses should develop a caring behaviour and compassion in dealing with patients. The development of these traits should be the focus of nursing education to guarantee caring and companionate future nurses.

However, nursing students are often faced with many challenges in their educational journey, such as academic and clinical stresses (Felicilda‐Reynaldo et al., 2017). Moreover, the learning environment, including teacher–student relationship and interaction, impacts the development of caring competencies of nursing students (Labrague et al., 2015). These challenges may pose risk to the students’ well‐being, thereby negatively affecting their delivery of competent and compassionate care to their patients (Alabdulaziz, Alquwez, Almazan, et al., 2020). Similarly, nursing students demonstrate less caring behaviour and compassion in clinical settings as reported in previous studies (i.e. Aboshaiqah et al., 2018; Alabdulaziz, Alquwez, Cruz, et al., 2020; Labrague et al., 2017). The value of self‐compassion on the topic of individual's well‐being has been discussed in previous studies. Self‐compassion has shown to affect one's psychological well‐being and self‐image (Alabdulaziz, Alquwez, Almazan, et al., 2020). Hence, self‐compassion of nursing students could be considered when examining the factors that might influence their caring behaviour and compassion competence due to its potential effect on the students’ well‐being, effective coping, emotional aspect and other personal outcomes (Neff, 2016). In addition, their competence in providing compassionate care is eventually affected. Therefore, we investigated the self‐compassion of student nurses on Saudi and examined its predictive role on their caring behaviour and compassion competence. The study hypothesized that the students’ self‐compassion predicts their caring behaviour and compassion competence.

## 2 BACKGROUND OF THE STUDY

Healthcare institutions worldwide are concerned about the limited holistic care core needs towards patient care delivery despite the advancement in delivering quality patient care (Sharp et al., 2018). For example, one study reported that the newly graduated nurses have difficulty in directly communicating and empathizing with patients’ clinical settings (Ebrahimi et al., 2016). In another study by Ingebretsen and Sagbakken (2016), nurses have difficulty in exhibiting compassion in providing nursing care to patients with unstable intense conditions (e.g. intense pain, distress and dying patients). The issue of patient dehumanization through a profession, which should promote humanistic care, is a serious concern and should be dealt with in the nursing profession (Głębocka, 2019).

Most certainly, the nurses’ caring and compassion competence are essential in providing nursing care. Most patients believed that the quality of patient care delivered to them depended on the compassion that nurses provided (Lee & Seomun, 2016a). Caring, on the other hand, is the core of nursing driven by authentic concern and love (Smith, 2014). Caring can either be physical or expressive. According to Labrague et al. (2017), the physical aspect of care is task‐oriented (e.g. pain management and medication administration) and the expressive aspect of care involves intervention activities, such as being with the client, providing emotional support and listening to the client. Evidence supports that patient's outcomes, such as satisfaction, effective coping and recovery, are positively influenced by patients’ encounter of effective caring behaviour from nurses, but defective caring may cause deterioration in the quality of care (Chana et al., 2015; Labrague et al., 2019). In summary, nurses can provide quality nursing care to patients in a compassionate manner when they are aware about the factors influencing caring behaviour and compassion competence.

However, caring could not be achieved without compassion. Compassion is the profound awareness and strong determination to assist an individual to be free from suffering (Chochinov, 2007). Compassion competence entails enthusiastically responding to patient needs, considering the patients’ physical, emotional and psychological strains (Dewar & Christley, 2013). From a nurse's perspective, compassion competence is a significant factor that influences their satisfaction level in performing nursing care (Lee & Seomun, 2016a). Compassionate care entails that nurses enter into the patients’ experience and ensure that patients maintain their independence and dignity (Lee & Seomun, 2016a). For nursing students, a compassionate care involves empathy, alleviation of the suffering of the patients, attending to individualized nursing needs, utilizing therapeutic communication and stimulating benefits of the patient and the nurse (Su et al., 2020). Previous research reported that being compassionate enhances patient quality care and the nurse's well‐being (Pattison & Samuriwo, 2016). However, in the recent years, nurses often experience compassion fatigue and become less compassionate in providing care. In addition, calls to enhance compassion among nurses have been emphasized in previous investigations (Nolte et al., 2017; Papadopoulos et al., 2016).

Several factors and concepts are crucial for nurses to develop caring behaviour and to become compassionate (Cruz et al., 2018). Among these factors, self‐compassion is theorized to impact nurses’ caring behaviour and compassion competence (Duarte et al., 2016). Self‐compassion is the capability to relate to own feelings and welfare (Mills et al., 2015). Self‐compassion is a stance of warmth, caring and empathy directed towards the self. Individuals with self‐compassion manifest more affection, humanity and altruism (Duarte et al., 2016; Neff & Pommier, 2013). An individual without self‐compassion has poor compassion competence and less care for others. Furthermore, self‐compassion is the capability to handle sorrow‐ and distress‐associated emotions (Lee & Seomun, 2016a). Notably, self‐compassion is not only crucial for nurse's outstanding patient care, but also for nursing students because they have responsibilities of dealing with academic work and clinical work instantaneously.

However, although investigation on self‐compassion and compassion competence creates momentum, limited literature has been conducted among nursing students, most especially in the Arab region. In addition, certain studies have been carried out on factors affecting nursing caring behaviour (Taylan et al., 2020; Malekzadeh et al., 2018), and no detailed investigation on the effect of self‐compassion on caring behaviour and compassion competence among nursing students has been conducted. To appraise and enhance the patient quality care rendered, exploring the self‐compassion of future nurses is meaningful. Hence, this research is timely to investigate the viewpoint of nursing students in Saudi Arabia regarding self‐compassion and examine its influence on their caring behaviour and compassion competence.

## 3 METHODS

This multiuniversity study utilized the quantitative design to survey student nurses from five Saudi universities. The universities have similar four‐year nursing programme with an additional year for internship. A convenience sampling technique was utilized with the following inclusion criteria: (a) second, third and fourth year nursing students and nursing interns; (b) have had or having clinical duties, and (c) males or females. First year nursing students were not surveyed because of their lack of nursing courses and clinical duties. A total of 1,200 students were invited to participate, and 961 nursing students agreed to participate and were included in the analyses. Hence, the response rate was 80.1%.

### 3.1 Ethical considerations

The Institutional Review Board of Majmaah University (MUREC‐ Apr.03 ICOM‐2019124) approved the study. The potential participants were informed about the study, their right to voluntarily participate and their required participation. They were also informed that the risk related to the study was minimal. Their performance in any class will not be affected by their participation. The students were encouraged to ask questions about the study. An informed consent was signed by each respondent. No incentives were offered for participation. A researcher, who is unrelated with the students, was responsible for data collection to avoid undue influence and potential coercion. Permission to use the tools in this study was granted.

### 3.2 Instruments

#### 3.2.1 Demographic variables

The demographic variables included age, gender, university, year of study, family structure and living environment.

#### 3.2.2 Self‐compassion scale (SCS)

This scale with 26 items and with a five‐point Likert response option was created by Neff (2003) to assess the self‐compassion of an individual along with six dimensions. The three dimensions pertain to positive self‐compassion behaviour (“Self‐kindness” with five items, “Common Humanity” with four items and “Mindfulness” with four items), and the three other dimensions focus on negative self‐compassion behaviour (“Self‐judgment” with five items, “Isolation” with four items and “Over‐identification” with four items). Subscale scores and an overall score were obtained by computing the means of the items after reversing the items in “Self‐judgment,” “Isolation,” and “Over‐identification.” Higher mean scores signify more positive self‐compassion. The scale's validity and reliability were well established by Neff (2003). The Cronbach's alpha for the entire scale was 0.92. For its subscales, the Cronbach's alpha are the following: 0.78 for “Self‐kindness,” 0.77 for “Self‐judgment,” 0.80 for “Common humanity,” 0.79 for “Isolation,” 0.75 for “Mindfulness” and 0.81 for “Over‐identification” (Neff, 2003). The Arabic version of the tool (SCS‐A) has acceptable validity and reliability when used among Saudi nursing students (Cronbach's *α* = 0.86). For the subscales of the SCS‐A, the Cronbach's alpha are the following: 0.80 for “Self‐kindness,” 0.83 for “Self‐judgment,” 0.85 for “Common humanity,” 0.79 for “Isolation,” 0.85 for “Mindfulness” and 0.76 for “Over‐identification” (Alabdulaziz, Alquwez, Almazan, et al., 2020). The overall Cronbach's alpha in this study was 0.66.

#### 3.2.3 Compassion competence scale (CCS)

This 17‐item tool, with five‐point “strongly disagree” (1) to “strongly agree” (5) response options, was created to screen nurses’ compassion competence in the clinical area (Lee & Seomun, 2016c). The CCS measures three subdomains of compassion competence, namely, “communication” (eight items), “sensitivity” (five items) and “insight” (four items). Mean dimension scores are calculated to reflect the level of compassion competence of the respondents along the three subdomains. A total mean score is also computed to reflect the overall compassion competence. The original version of the scale demonstrated excellent validity and reliability as previously reported. The Cronbach's alpha for the whole scale was 0.91, while 0.88, 0.77 and 0.73 were computed for “communication,” “sensitivity” and “insight,” respectively (Lee & Seomun, 2016c). The Arabic version (CCS‐A) by Alabdulaziz, Alquwez, Cruz, et al. (2020), with acceptable validity and reliability for Saudi nursing students, was used in this study. The Cronbach's alpha of the Arabic version was 0.806, and the EFA established the three subdomains accounting for 50.6% of the compassion competence variance of Saudi nursing students. For its subscales, a Cronbach's alpha of 0.797, 0.788 and 0.739 were computed for “communication,” “sensitivity” and “insight,” respectively (Alabdulaziz, Alquwez, Cruz, et al., 2020). The overall Cronbach's alpha in this study was 0.88.

#### 3.2.4 Caring behavior inventory (CBI)

The CBI‐16 version of the CBI by Wolf et al., (2017) is adapted in this study. The CBI was grounded on Watson's theory of caring and was originally developed to assess the perceptions of patients towards the caring behaviour of nurses (Wolf et al., 1994). The CBI‐16 is responded using a six‐point Likert scale from 1 (never) to 6 (always). Scores can be obtained by summing the individual score of each item in the scale (possible score range = 16–96), with higher scores implying better caring behaviour. The factor analysis revealed that the CBI‐16 has one factor with explained variance of 58.0%. The computed Cronbach's alpha of the CBI‐16 version was 0.95, indicating excellent internal consistency (Wolf et al., 2017).

### 3.3 Validity and reliability of the CBI‐16 Arabic version (CBI‐16‐A)

The CBI‐16 was translated to Arabic language following the forward–backward translation method. Two Saudi assistant professors in nursing separately translated the tool to Arabic language. Another Saudi assistant professor in nursing synthesized the two translations to create a single version, which was presented and approved by the two translators. Then, the tentative Arabic version was translated back separately to English by two translators. The tentative Arabic version and the back‐translated versions were presented to a panel of experts with five members, who evaluated the equivalency of the different versions. Once the panel had agreed on the Arabic version (CBI‐16‐A), content validity was then computed. The item‐level and scale‐level content validity indices were within the acceptable level, indicating excellent content validity. The CBI‐16‐A was distributed to a convenience sample of 160 Saudi student nurses from a public university in Riyadh, Saudi Arabia. These students were excluded from the present study. The computed Cronbach's alpha of the tool based on the data from the 160 sample was 0.961, which signifies excellent internal consistency of the tool. The EFA revealed a single factor of the tool accounting for 67.0% for the variance (factor loadings = 0.65 to 0.93). This finding indicates the acceptable construct validity of the tool when used among Saudi nursing students.

### 3.4 Data collection

The researchers approached the qualified students during their break time to explain about the study. Those who signed the inform consent were provided with the survey. The students were instructed to drop the answered questionnaire in the boxes positioned in different places in the universities. At the end of each week, the researchers collected the questionnaires from the drop boxes until the data collection period ends. The data collection was conducted on August to November 2019.

### 3.5 Data Analysis

Descriptive analyses were conducted in the demographic variables. Mean and standard deviation were calculated for all variables (self‐compassion, caring behaviour, and compassion competence). T tests, one‐way ANOVA with Tukey HSD test, and Pearson's correlations were carried out to investigate the relationship between the self‐compassion and demographics. Standard multiple analysis was performed separately for caring behaviour and compassion competence as dependent variables. For both models, the self‐compassion dimensions and the demographic variables were considered predictor variables. Dummy variables were created for the variable university and year level prior to use in the regression model. P‐values less than 0.05 denoted statistical significance. The SPSS version 22.0 was utilized for all the analyses.

## 4 RESULTS

Data from 961 Saudi nursing students were included in the analyses. The ages of the students were from 18 to 38 years with a mean of 22.27 (*SD* = 3.19). Most respondents were females (52.0%), with a nuclear family (69.5%) and urban dwellers (77.2%). The highest proportion of the sample was from University 2 (31.1%), followed by Universities 3 (22.2%), 5 (16.8%) and 1 (16.4%). The lowest sample proportion was from University 4 (13.5%). In terms of the year level, the highest sample was from the second year students (36.6%), followed by fourth year students (27.1%), third year students (21.1%) and then nursing interns (15.2%; Table 1).

**TABLE 1:** The demographic characteristics of the respondents (*n* = 961)

Demographic variables	Mean (*SD*)	Range
Age	22.27 (3.19)	18–38
Gender	*n*	%
Male	461	48.0
Female	500	52.0
University		
University 1	158	16.4
University 2	299	31.1
University 3	213	22.2
University 4	130	13.5
University 5	161	16.8
Year level		
2nd year	352	36.6
3rd year	203	21.1
4th year	260	27.1
Internship	146	15.2
Type of family		
Nuclear family	668	69.5
Extended family	293	30.5
Type of community		
Rural	219	22.8
Urban	742	77.2

### 4.1 Self‐compassion and its associated demographic variables

The overall mean score in the SCS was 3.15 (*SD* = 0.38). The highest mean was recorded in the subscale “mindfulness” (*M* = 3.35, *SD* = 0.79), followed by the subscales “self‐kindness” (*M* = 3.34, *SD* = 0.74), “common humanity” (*M* = 3.24, *SD* = 0.72), “over‐identification” (*M* = 3.09, *SD* = 0.73), “isolation” (*M* = 2.97, *SD* = 0.78) and “self‐judgment” (*M* = 2.92, *SD* = 0.79; Table 2).

**TABLE 2:** Results of the descriptive analyses on the research variables (*n* = 961)

Variable	Range		Mean	*SD*
Self‐compassion	1.88	4.54	3.15	0.38
Self‐Kindness	1.00	5.00	3.34	0.74
Self‐Judgment	1.00	5.00	2.92	0.79
Common Humanity	1.00	5.00	3.24	0.72
Isolation	1.00	5.00	2.97	0.78
Mindfulness	1.00	5.00	3.35	0.79
Over‐Identification	1.00	5.00	3.09	0.73
Caring behaviour	1.00	6.00	4.76	0.95
Compassion Competence	1.00	5.00	3.73	0.64
Communication	1.00	5.00	3.59	0.80
Sensitivity	1.00	5.00	4.04	0.76
Insight	1.00	5.00	3.62	0.76

As shown in Table 3, age and self‐compassion had a very weak positive correlation (*r* = 0.07, *p* = .023). Significant differences were observed on the students’ self‐compassion between universities (*F* = 25.71, *p* < .001). The Tukey HSD test revealed that the reported self‐compassion among nursing students from Universities 1 and 2 was statistically lower than the reported self‐compassion of students from Universities 3, 4 and 5. Moreover, students from University 3 reported statistically lower self‐compassion than students from University 4. These findings indicate that students from Universities 1 and 2 had poorer self‐compassion than the students from the other universities. On the contrary, students from University 4 had more positive self‐compassion than the others.

**TABLE 3:** Results of the tests of association between the respondents’ demographic characteristics and self‐compassion (*n* = 961)

Demographics	Mean	*SD*	Statistical test	*p*
Age			*r* = 0.07	.023*
Gender				
Male	3.15	0.35	*t* = 0.18	.854
Female	3.15	0.40		
University^a^				
University 1	3.04	0.21	*F* = 25.71	<.001***
University 2	3.03	0.25		
University 3	3.19	0.45		
University 4	3.35	0.47		
University 5	3.25	0.45		
Year level				
2nd year	3.14	0.38	*F* = 1.99	.114
3rd year	3.11	0.33		
4th year	3.17	0.40		
Internship	3.20	0.38		
Type of family				
Nuclear family	3.15	0.39	*t* = −0.43	.667
Extended family	3.16	0.35		
Type of community				
Rural	3.14	0.34	*t* = −0.19	.861
Urban	3.15	0.39		

^a^ University 1 versus University 3 (*p* = .001), University 1 versus University 4 (*p* < .001), University 1 versus University 5 (*p* < .001); University 2 versus University 3 (*p* < .001), University 2 versus University 4 (*p* < .001), University 2 versus University 5 (*p* < .001); University 3 versus University 4 (*p* = .001).

* Significant at 0.05 level.

*** Significant at 0.001 level.

### 4.2 Caring behaviour and the influence of self‐compassion

The mean score in the CBI‐16‐A was 4.76 (*SD* = 0.95; Table 2). The regression model was statistically significant (*F*[17, 943] = 22.97, *p* <.001), explaining approximately 28.0% of the variance of the students’ caring behaviour (*R* ^2^ = 0.293, Adjusted *R* ^2^ = 0.280). Among the predictor variables in the model, three demographic variables (university, year level and type of family) and two self‐compassion subscales (“self‐kindness” and “common humanity”) were identified as significant predictors of caring behaviour. As reflected in Table 4, students from University 1 reported significantly poorer caring behaviour than students from Universities 2 (*β* = 1.19, *p* < .001, 95% Confidence Interval [CI] = 1.00, 1.37), 3 (*β* = 0.98, *p* < .001, 95% CI = 0.78, 1.17), 4 (*β* = 0.91, *p* < .001, 95% CI = 0.71, 1.11) and 5 (*β* = 1.18, *p* < .001, 95% CI = 0.98, 1.39). Nursing interns had better caring behaviour than the second year students (*β* = 0.18, *p* = .039, 95% CI = 0.01, 0.36). Students belonging to an extended type of family had better caring behaviour than those from nuclear family (*β* = 0.15, *p* = .010, 95% CI = 0.04, 0.27). For the self‐compassion subscales, a point increase in the “self‐kindness” mean score corresponded to 0.16 point (*p* < .001, 95% CI = 0.07, 0.26) increase in the caring behaviour mean score. Moreover, a point increase in the “common humanity” mean score caused 0.22 point (*p* < .001, 95% CI = 0.13, 0.30) increase in the caring behaviour mean score.

**TABLE 4:** Results of the multiple regression analysis on the nursing students’ caring behaviour (*n* = 961)

Predictor variable	*β*	*SE*‐b	Beta	*t*	*p*	95% Confidence Interval	
						Lower	Upper
Age	0.00	0.01	0.00	−0.11	.909	−0.02	0.02
Gender	−0.06	0.06	−0.03	−0.95	.342	−0.17	0.06
University (Reference: University 1)							
University 2	1.19	0.09	0.58	12.68	<.001***	1.00	1.37
University 3	0.98	0.10	0.43	9.84	<.001***	0.78	1.17
University 4	0.91	0.10	0.33	8.85	<.001***	0.71	1.11
University 5	1.18	0.11	0.47	11.27	<.001***	0.98	1.39
Year level (Reference: 2nd year)							
3rd year	0.11	0.07	0.05	1.48	.139	−0.04	0.25
4th year	0.14	0.07	0.07	1.95	.051	0.00	0.28
Internship	0.18	0.09	0.07	2.07	.039*	0.01	0.36
Type of family	0.15	0.06	0.07	2.58	.010*	0.04	0.27
Type of community	−0.04	0.07	−0.02	−0.52	.597	−0.17	0.10
Self‐compassion subscales							
Self‐Kindness	0.16	0.05	0.13	3.50	<.001***	0.07	0.26
Self‐Judgment	−0.06	0.05	−0.05	−1.10	.270	−0.15	0.04
Common Humanity	0.22	0.04	0.16	4.86	<.001***	0.13	0.30
Isolation	0.02	0.05	0.02	0.47	.642	−0.08	0.12
Mindfulness	0.06	0.05	0.05	1.40	.163	−0.03	0.15
Over‐Identification	0.04	0.05	0.03	0.86	.391	−0.05	0.13

Note

The dependent variable was the overall mean of the Caring Behavior Inventory. *β* is the unstandardized coefficients; *SE*‐b is the Standard error.

*R*^2^ = 0.293, Adjusted *R* ^2^ = 0.280

* Significant at 0.05 level.

*** Significant at 0.001 level.

### 4.3 Compassion competence and the influence of self‐compassion

In Table 2, the mean score in the CCS‐A was 3.73 (*SD* = 0.64). The highest mean was recorded in the subscale “sensitivity” (*M* = 4.04, *SD* = 0.76), followed by “insight” (*M* = 3.62, *SD* = 0.76) and “communication” (*M* = 3.59, *SD* = 0.80). The regression model was statistically significant (*F*[17, 943] = 26.19, *p* < .001), explaining approximately 30.8% of the variance on the students’ compassion competence (*R* ^2^ = 0.321, Adjusted *R* ^2^ = 0.308). As shown in Table 5, university, level, type of family, “self‐kindness,” “self‐judgment,” “common humanity,” and “mindfulness” were significant predictors of compassion competence. Student nurses attending University 1 had lower levels of compassion competence than students from Universities 2 (*β* = 0.47, *p* < .001, 95% CI = 0.35, 0.59), 3 (*β* = 0.72, *p* < .001, 95% CI = 0.59, 0.85), 4 (*β* = 0.43, *p* < .001, 95% CI = 0.29, 0.56) and 5 (*β* = 0.53, *p* < .001, 95% CI = 0.39, 0.66). Students in the fourth year recorded 0.11 point higher compassion competence score than students from the second year (*p* = .021, 95% CI = 0.02, 0.20). However, students belonging to an extended type of family recorded 0.10 point higher compassion competence score than students belonging to a nuclear family (*p* = .012, 95% CI = 0.02, 0.17). Moreover, a point increase in the mean scores of “self‐kindness,” “common humanity,” and “mindfulness” resulted to 0.11 point (*p* < .001, 95% CI = 0.05, 0.17), 0.12 point (*p* < .001, 96% CI = 0.07, 0.18) and 0.15 point (*p* < .001, 95% CI = 0.10, 0.21) increase in the compassion competence mean score. On the contrary, a point increase in the “self‐judgment” mean score reflected 0.09 point (*p* = .010, 95% CI = −0.15, −0.02) decrease in the compassion competence mean score.

**TABLE 5:** Results of the multiple regression analysis on the nursing students’ compassion competence (*n* = 961)

Predictor variable	*β*	*SE*‐b	Beta	*t*	*p*	95% Confidence Interval	
						Lower	Upper
Age	−0.01	0.01	−0.05	−1.43	.153	−0.02	0.00
Gender	0.04	0.04	0.03	1.02	.306	−0.04	0.12
University (Reference: University 1)							
University 2	0.47	0.06	0.34	7.62	<.001***	0.35	0.59
University 3	0.72	0.07	0.47	10.98	<.001***	0.59	0.85
University 4	0.43	0.07	0.23	6.30	<.001***	0.29	0.56
University 5	0.53	0.07	0.31	7.62	<.001***	0.39	0.66
Year level (Reference: 2nd year)							
3rd year	0.05	0.05	0.03	0.96	.338	−0.05	0.14
4th year	0.11	0.05	0.08	2.31	.021*	0.02	0.20
Internship	0.03	0.06	0.02	0.56	.578	−0.08	0.15
Type of family	0.10	0.04	0.07	2.51	.012*	0.02	0.17
Type of community	−0.04	0.05	−0.03	−0.89	.371	−0.13	0.05
Self‐compassion subscales							
Self‐Kindness	0.11	0.03	0.13	3.67	<.001***	0.05	0.17
Self‐Judgment	−0.09	0.03	−0.11	−2.60	.010*	−0.15	−0.02
Common Humanity	0.12	0.03	0.14	4.27	<.001***	0.07	0.18
Isolation	−0.02	0.03	−0.02	−0.53	.599	−0.08	0.05
Mindfulness	0.15	0.03	0.19	5.17	<.001***	0.10	0.21
Over‐Identification	0.01	0.03	0.02	0.46	.643	−0.05	0.08

Note

The dependent variable was the overall mean of the Compassion Competence Scale. *β* is the unstandardized coefficients; *SE*‐b is the Standard error.

*R*^2^ = 0.321, Adjusted *R* ^2^ = 0.308

* Significant at 0.05 level.

*** Significant at 0.001 level.

## 5 DISCUSSION

This study investigated the Saudi nursing students’ self‐compassion and examined its predictive role on their caring behaviour and compassion competence. An overall modest self‐compassion was exhibited by the Saudi nursing students in this study. Comparing this finding to previous studies, nursing students in Turkey (Hiçdurmaz & Aydin, 2017) reported better self‐compassion, but students in the United States and in Taiwan reported slightly poorer self‐compassion (Neff et al., 2008). Self‐compassion has been defined in this study as “compassion turned inward and refers to how we relate to ourselves in instances of perceived failure, inadequacy or personal suffering” (Neff, 2016, p. 265). The tool used to measure self‐compassion was designed around three main ideas, each has a positive and negative end that characterizes compassionate and uncompassionate behaviour. The modest self‐compassion of nursing students in this study may imply their ambivalent perceptions and feelings towards determining compassionate and uncompassionate behaviour. This finding could be supported by the subscale scores wherein compassionate behaviour (mindfulness, self‐kindness and common humanity) was perceived more positively than uncompassionate behaviour (over‐identification, isolation and self‐judgment). For example, some students may have implied clear and balanced awareness of the sufferings that they currently experience (mindfulness), but some may indulge in overstated storyline of negative features of their experiences (over‐identification). Similarly, some students may be gentle, supportive and understanding to themselves (self‐kindness), but some may be too harsh to themselves whenever they experience difficulties in life (self‐judgment). Hiçdurmaz and Aydin (2017) argued that one's self‐compassion could be influenced by culture. This finding was illustrated in a previous study among undergraduate students by Neff et al. (2008), where Taiwanese students’ high self‐judging and avoidance of negative emotions influenced their poor self‐compassion. On the contrary, Thai students’ positive approach to life's failures and difficulties resulted in their high self‐compassion. Nonetheless, nursing education should include the importance of nourishing the self‐compassion of nursing students.

Some students’ demographic variables were associated with self‐compassion in this study. Specifically, age and self‐compassion are positively related. This finding implies that older students have greater perceived self‐compassion. A possible explanation is the age‐related development that as one gets older, more experiences in dealing with life issues are obtained. Other empirical data reported that older nursing students had more experience with various work‐related circumstances, which could develop better relationships with patients and their families, and nurture compassion (Alshehry et al., 2019). Varying levels of self‐compassion were also reported among students from different universities in this study. However, further investigations are required to determine the specific academic‐related factors that affect the students’ self‐compassion.

Our data showed that the nursing students in Saudi had modest caring behaviour, which is considered lower than those reported by nursing students in Iran (Azizi et al., 2013), Philippines and Nigeria (Labrague et al., 2017), but higher than the multicountry study conducted in Greece, India (Labrague et al., 2017) and Singapore (Loke & Lee, 2016). This result affirms the satisfactory caring behaviour of nursing students in Saudi in a recent study (Allari et al., 2020). The concept of caring within the Arab context is grounded on the premise that “caring is a spiritual action.” According to Lovering (2012), the essence of caring among Muslim nurses begins with their relationship with Allah (God) and is built around the teachings of Islam. Hence, nurses’ caring actions to patients are rewarded by Allah. This belief has been ingrained among Saudis starting from the very young age. However, the development of caring behaviour among nursing students is influenced by various factors. For example, a previous study presented that nursing students’ attitudes, plans and nursing experiences influenced their caring behaviour (Konuk & Tanyer, 2019). Moreover, Labrague et al., (2015) concluded that the caring behaviour of nursing students could be positively influenced by their instructors’ positive caring behaviour. Hence, nursing instructors could assist in developing their students’ caring behaviour through positive role modelling.

Another highlight of the study is the moderate level of compassion competence reported by the nursing students; it was slightly lower than those reported in a multicountry study involving Iraq, Nigeria, Oman and South Korea (Samson‐Akpan et al., 2019). Our data also show the highest competence on the ability of the students to determine and respond to the emotional changes experienced by the patients. A multicountry study recorded similar findings, where nursing students from Iraq, Nigeria, Oman and South Korea perceived the highest competence in being sensitive to patient's emotions (Samson‐Akpan et al., 2019). However, a previous review study among healthcare professionals and healthcare students emphasized their poor compassion competence because of their exposure to complex patients’ conditions and the availability and use of various sophisticated and advanced technology that often dehumanize patient care (Sinclair et al., 2017). Hofmeyer et al. (2016) discovered that nursing students have low compassion competence because of difficult and demanding patients, substandard working conditions and overwhelmingly unconstructive clinical culture, thereby leading to negative self‐judgment, stress, compassion fatigue and negative well‐being.

Further findings of the study revealed that year level was identified as significant predictors of caring behaviour and compassion competence. Specifically, nursing interns and senior students had better caring behaviour and greater compassion competence, respectively, compared with sophomores. These results could be explained by the progression of knowledge, skills and attitudes of nursing students as they climb to the nursing educational ladder as a result of their theoretical learning in the major nursing courses and their actual experience of providing care to patients. Internship programmes are usually offered by healthcare organizations to nursing students in their final year. These programmes provide learning opportunities through direct patient care and immersion to the clinical environment, which is beneficial in competency development (Ahmadi et al., 2020). The final year presents more opportunities to exhibit great levels of caring behaviour, and lower year levels offer limited caring experience and compassion competency among students (Konuk & Tanyer, 2019). Another important finding is the influence of the university, wherein varying levels of caring behaviour and compassion competence were observed between students from different universities. This finding may imply the differences in developing these competencies in each nursing programme in the country. The lack of a unified nursing curriculum among different universities in the country may have contributed to these differences and, thus, calls for a development of a national unified nursing curriculum and nursing competencies in the country (Aljohani, 2020).

Results in this study also exhibited that students belonging to an extended family had better caring behaviour and compassion competence than students from a nuclear family. This finding is worth noting because of the potential role of families in aiding the development of caring and compassion competencies of an individual (Al‐Khraif et al., 2020). Alshehry et al. (2019) suggested that an extended family assures that individuals spend more time with other members (e.g. grandparents), thereby nurturing a caring environment and eventually enhancing the caring behaviour and sense of compassion of the family members.

“Self‐kindness” and “common humanity” positively influenced the caring behaviour of student nurses. An individual, who embodies “self‐kindness,” is gentle, supportive and understanding towards self at times of struggles, challenges and difficulties (Neff, 2016). Neff (2016) discussed that having common humanity encompasses appreciating shared human experiences, acknowledging that life is imperfect, and understanding that failures and mistakes are part of human life. Previous studies provided evidence on the positive impact of “self‐kindness” and “common humanity” on nursing students and nurses’ emotional intelligence (Şenyuva et al., 2014), mental health (Luo et al., 2019) and professional quality of life (Duarte et al., 2016). These variables are essential factors that might influence a nurses’ ability in providing care to patients, which could thereby explaining the role of self‐respect on caring behaviour. Moreover, people who are kind to themselves and have sense of common humanity are happy (Neff & Pommier, 2013), thereby enabling them to be kind to others and to create positive social connections with others. This condition could lead them to be more caring to others. Accepting the imperfections of life, appreciating shared human experiences and being kind to one's self during difficult life's experiences may assist nursing students to understand the situation and suffering of their patients; thus, they could show more care for them better.

Furthermore, “self‐kindness,” “common humanity,” and “mindfulness” positively influence compassion competence, whereas “self‐judgment” negatively influences compassion competence among the nursing students in Saudi. These findings echo the findings of previous studies claiming that having positive self‐compassion among healthcare providers positively impacts their compassion competence (Sinclair et al., 2017). Ying (2009) further stressed that when people are mindful about themselves and their situations, they become more compassionate and less judgmental. Individuals with greater self‐compassion are more flexible in life, can easily adjust in different life situations and have tendencies to be more connected with others (Neff & Pommier, 2013). Durkin et al., (2016) argued that when nurses are compassionate towards themselves, they are more likely to exhibit greater compassion towards their patients. Our findings also support the five essential elements of compassion, as follows: (a) recognizing suffering, (b) accepting suffering as a universal human experience, (c) being empathetic to the suffering of others, (d) understanding uncomfortable feelings when responding to a suffering individual and (e) being motivated to act to relieve suffering (Strauss et al., 2016). Barrat (2017) argued that self‐compassion is necessary to achieve these elements of compassion towards others. For instance, to facilitate recognition of other people's suffering, individuals must be aware and accept themselves and their own sufferings. Moreover, element two requires common humanity, which is the ability to accept that failures and suffering are part of life. Self‐compassion is also required to form connection with the suffering of the patients and enables them to provide appropriate responses to these sufferings. These findings could explain the influence of self‐compassion on compassion competence among the nursing students.

However, this study has some limitations that need to be considered. Despite the multiuniversity nature of the study, it was only conducted in government universities and no private universities were included. Hence, the findings may not represent the perceptions of students in private universities. In addition, the use of convenience sample limits the generalizability of the findings. Another limitation is the self‐report nature of the variables, thereby causing a certain level of social desirability bias. However, the high response rate and sample size may have augmented this weakness.

## 6 CONCLUSIONS AND IMPLICATIONS

Our study provides evidence on the positive influence of self‐compassion on caring behaviour and compassion competence among nursing students in Saudi. Self‐compassion, caring behaviour and compassion competence vary significantly among students from different universities. Being in the higher years of the nursing programme and belonging to an extended family positively affect the student nurses’ caring behaviour and compassion competence.

This investigation underscores the significance of self‐compassion in the development of caring behaviour and compassion competence of student nurses. The results advocate the need to nurture the nursing students’ self‐compassion while they go through their journey in nursing education. Nursing education must realize the value of self‐compassion and integrate educational interventions in the curriculum that foster its development. Learning activities, in the classroom and in the clinical settings, should be incorporated with lessons about self‐compassion, particularly on strengthening the compassionate behaviour and controlling uncompassionate behaviour. Moreover, to develop a better plan and implementation of such educational interventions, constant assessment of each student's self‐compassion and identification of the areas to be strengthened and factors that impact the development of self‐compassion should be conducted. This approach will enable an individualized approach in developing the students’ self‐compassion. The present findings provide these baseline data that could be used in initial planning and implementation of such interventions. Ultimately, the study emphasizes the development of caring behaviour and compassion competence among nursing students by tailoring the curriculum to foster caring and compassion concepts during their early nursing years and providing more learning opportunities, where students could create a more positive compassion towards self.

## CONFLICT OF INTEREST

Dr. Jonas Preposi Cruz is a member of the Editorial Board of the journal. The other authors declare no conflict of interest.

## AUTHORS’ CONTRIBUTION

Conception and design: all authors. Acquisition of data: all authors. Analysis and interpretation of data: NA, JPC, AAT. Drafting the manuscript: NA, JPC, JA. Revising the manuscript critically for important intellectual content. Given final approval of the version to be published: all authors. Agreed to be accountable for all aspects of the work: all authors.

## Data Availability

The data that support the findings of this study are available from the corresponding author upon reasonable request.
